# Hematological Characteristics of Patients With Sickle Cell Disease in Al Ahsa, Saudi Arabia

**DOI:** 10.7759/cureus.87432

**Published:** 2025-07-07

**Authors:** Mohammad H Al Khamees, Bader AlAlwan, Mohammed Al Saleh, Aymen A Alqurain, Mohammed S Bu-Kheder, Habeeb K Bin Khalaf, Yahya A Al Fenes, Baqer Alkhadhrawi, Abdulhadi Al Hajji, Nida Alsafar

**Affiliations:** 1 Laboratory Medicine, King Fahad General Hospital, Al Hofuf, SAU; 2 Laboratory Medicine, Mohammed Al-Mana College for Medical Sciences, Dammam, SAU; 3 Pharmacy, Mohammed Al-Mana College for Medical Sciences, Dammam, SAU

**Keywords:** al ahsa, anemia, hematological characteristics, saudi arabia, scd, sickle cell crisis

## Abstract

Sickle cell disease (SCD) is an autosomal recessive illness characterized by severe morbidity and mortality rates. Although numerous studies on SCD have been conducted worldwide, the hematological features of SCD in the Middle East require further investigation. Therefore, we conducted this study to examine the hematological characteristics of SCD in Saudi Arabia, specifically in the Al Ahsa region. We performed a retrospective examination of health records at King Fahad Hospital in Hofuf, and data were analyzed in relation to gender, age, and other demographic factors. This study included a total of 96 records, with an average age of patients being 33 years. The majority of the patients (60%) were male. Most of the patients were anemic (99%), with one-quarter (26%) suffering from mild-to-moderate anemia (hemoglobin (Hb) above 10 g/dL) and the remaining three-quarters (74%) suffering from severe anemia (Hb below 10 g/dL). Correlation studies show that older patients (above 50 years old) are more severely affected by the disease compared to younger adult groups, evidenced by a strong negative correlation between age and both Hb levels and red blood cell counts (RBCs). Severe anemia is more common in females than in males, with a mean Hb of 8.4 g/dL in females (79% having Hb below 10 g/dL) compared to a mean Hb of 9.0 g/dL in males (71% having Hb below 10 g/dL). About half of the studied SCD population showed abnormal platelet counts (51%), with no significant difference between males and females. Thrombocytosis was slightly more prevalent than thrombocytopenia (31% vs. 20%). Our study indicates that SCD in Al Ahsa, Saudi Arabia, is associated with moderate-to-severe anemia. The impact of the disease is more pronounced in older individuals and females. Abnormal platelet counts are common among SCD patients in the Al Ahsa region. These findings should be taken into account when addressing SCD in the eastern province of Saudi Arabia and possibly in other regions.

## Introduction

Sickle cell disease (SCD) is a growing global health concern that shows an increasing number of birth of babies born with the disease by 13.7% from 2000 to 2021, with 376,000 total deaths estimated globally in 2021. The prevalence in the United States is reported to be as high as 329 cases per 1,000,000, while in Africa and the Middle East, the prevalence is reported to be higher, as it is estimated to be 20,000 per 1,000,000 in Nigeria and 45,100 per 1,000,000 in adults in Saudi Arabia [1].

SCD is an inherited blood disorder caused by a pathogenic substitution mutation in the β-globin gene that replaces the hydrophilic amino acid glutamine with the hydrophobic valine at the sixth position of the β-globin chain, resulting in the formation of a poorly soluble form of hemoglobin (Hb), Hb S. In a low oxygen state, Hb S polymerizes and becomes distorted and rigid, causing the red cells to change their morphology and show the characteristic of a sickle cell form [2,3]. There are several variations and genotypes of SCD, which range from homozygous SCD (SS) to sickle-hemoglobin C disease (SC) and sickle cell β-thalassaemia, among others, each with varying severity and clinical prognosis [1].

SCD has several complications, both acute and chronic, which translate to an unfavorable quality of life for the patients. SCD complications, including pain crisis, are usually either due to hemolysis or vaso-occlusive pathology [4]. These will manifest in the shape of a plastic or acute chest syndrome, strokes, deep vein thrombosis (DVT), and other vaso-occlusive crises. This adds to emotional and economic stress and the negative psychological impact of the disease on the life of the patients [5,6].

SCD affects millions of people around the world and is particularly common in West Africa, India, and the Middle East countries, including Saudi Arabia. Despite having the same genetic mutation among these populations, studies showed a significant variation in the hematological picture among these groups [7].

While the African and Indian variants were largely studied, less data are available about the Middle Eastern variant found in Al Ahsa, Saudi Arabia. The first documented case of SCD in Saudi Arabia was reported in 1963 in the eastern province [8]. The prevalence of the sickle cell gene in the adult population was estimated by the Saudi Premarital Screening Program to be 0.26% for SCD, with the highest prevalence in the eastern region, with 73% of deaths occurring under the age of 30, primarily due to acute chest syndrome [7].

Several attempts were made after that to elucidate the epidemiology and clinical phenotypes in different regions in Saudi Arabia [9,10]. However, more studies are still needed to understand the associated hematological pictures in certain areas in eastern provinces, such as in Al Ahsa region in Saudi Arabia, hence the need to undertake the current study.

## Materials and methods

Study design and data collection

This retrospective study was conducted to analyze hematological characteristics by reviewing the medical records of the patients admitted to the inpatient unit of King Fahad Hospital in Hofuf (KFHH), Saudi Arabia. The study period covered admissions from January to December 2021. The population of interest included adult patients of Saudi origin with confirmed SCD residing in Al Ahsa, Saudi Arabia. Data were extracted from the patients’ medical records and included demographic information (age and gender), treatment and management plans, and basic hematological laboratory results. To ensure confidentiality, all patient data were anonymized prior to access. The hospital's ethics committee approved the study and waived the need for informed consent.

Inclusion and exclusion criteria

Medical records of patients with confirmed diagnosis of SCD who were 18 years or older at the time of admission, were of Saudi origin, and were admitted in the duration of the study period (January to December 2021), while medical records with incomplete or unclear data or those for non-Saudi patients were excluded.

Sample size calculation

The sample size for the study was calculated to be 385 participants, determined by the Richard Geiger equation, with a margin of error determined as 5% and a confidence level of 95%. A convenient sampling technique was employed to identify and collect data from eligible participants.

Data management and statistical analysis

Data were collected and entered into a Microsoft Excel spreadsheet (Microsoft Corp., Redmond, WA), and subsequent statistical analysis was performed using the Statistical Product and Service Solutions (SPSS, IBM SPSS Statistics for Windows, Armonk, NY) software and GraphPad Prism (version 8; GraphPad Software, San Diego, CA). Descriptive statistics, including mean, median, and standard deviation, were calculated accordingly. Further statistical analysis was conducted to evaluate potential associations between age, gender, and various hematological parameters.

Ethical consideration

All data collected for this study were anonymized to ensure the confidentiality and privacy of participants and were used exclusively for research purposes. Ethical clearance was obtained from the Institutional Review Board (IRB) at KFHH prior to the initiation of the study. The study protocol was reviewed and approved by the hospital's ethical committee under registration number H-05-HS-065 and was granted IRB approval number 02-E-2024 on 29-01-2024. The research was conducted in full compliance with the ethical principles outlined in the Declaration of Helsinki and adhered to all relevant local and international guidelines for human subjects research.

## Results

**Figure 1:**
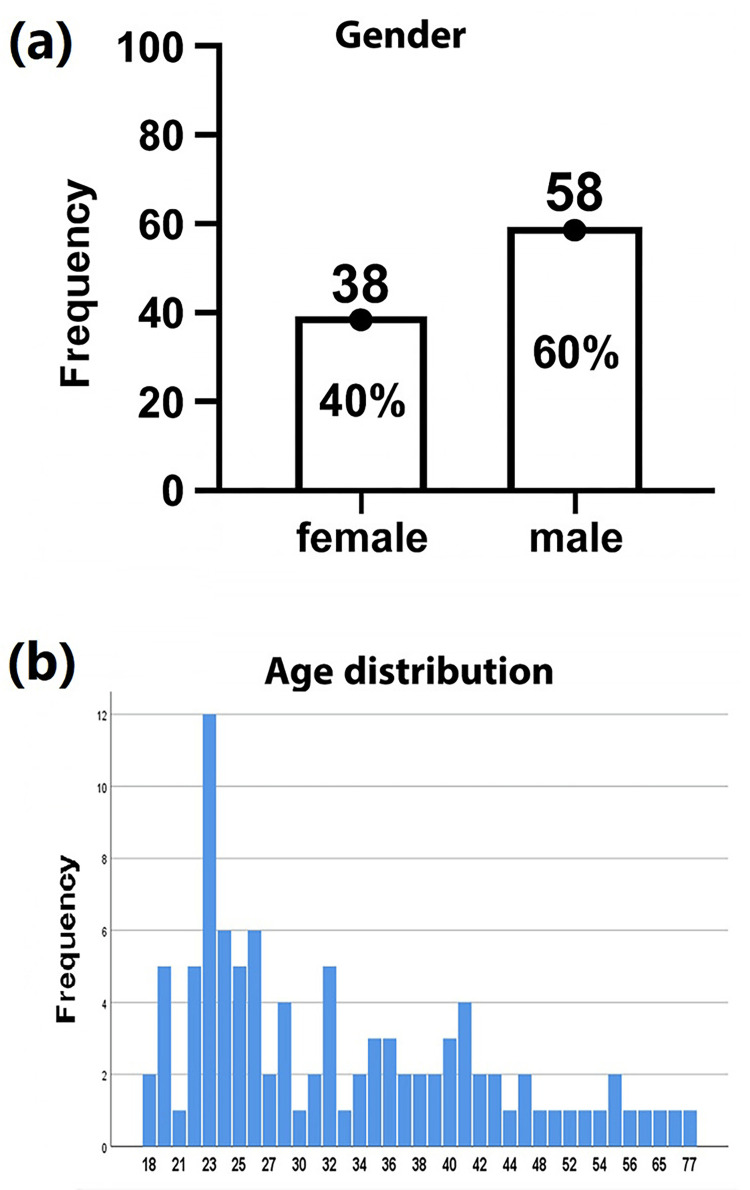
Age and gender distribution of the study population. (a) Gender distribution of the study participants showing 38 females (40%) and 58 males (60%) out of a total of 96 participants. (b) Age distribution histogram illustrating the frequency of participants across different age groups, with the highest concentration observed in the 22–26 age range.

Ninety-nine patients were initially included; however, three patients were later excluded for incomplete data. Therefore, only 96 patients were eventually included in this study. Fifty-eight of them were male (60%), while 36 (40%) were female patients. Most of the studied patients were young (18-50 years old), with a mean age of 33 and a maximum age of 77, as illustrated in Figure 1 and Tables 1-3.

**Table 1:** Descriptive statistics for the age of participants. The table includes the number of valid and missing entries, measures of central tendency (mean, median, mode), measures of dispersion (standard deviation, variance, range), and the minimum and maximum observed ages among the 96 participants.

N	Valid	96
	Missing	0
Mean		33.2083
Median		30.5000
Mode		23.00
Std. Deviation		12.18534
Variance		148.482
Range		59.00
Minimum		18.00
Maximum		77.00

**Table 2:** Pearson correlation between age and hemoglobin (HB) levels among the 96 participants. The table presents correlation coefficients, significance values (2-tailed), and the sample size. A significant negative correlation (r = -0.266, p = 0.009) was found, indicating that as age increases, HB levels tend to decrease. Correlation is significant at the 0.01 level (2-tailed).

		Age	HB
Age	Pearson Correlation	1	-0.266**
	Sig. (2-tailed)		0.009
	N	96	96
HB	Pearson Correlation	-0.266**	1
	Sig. (2-tailed)	0.009	
	N	96	96
** Correlation is significant at the 0.01 level (2-tailed).			

**Table 3:** Pearson correlation between age and red blood cell count (RBC) among the 96 participants. The table displays correlation coefficients, two-tailed significance levels, and sample sizes. A statistically significant negative correlation (r = -0.284, p = 0.005) was observed, indicating that RBC counts tend to decrease with increasing age. Correlation is significant at the 0.01 level (2-tailed).

		Age	RBC
Age	Pearson Correlation	1	-0.284**
	Sig. (2-tailed)		0.005
	N	96	96
RBC	Pearson Correlation	-0.284**	1
	Sig. (2-tailed)	0.005	
	N	96	96
** Correlation is significant at the 0.01 level (2-tailed).			

**Figure 2:**
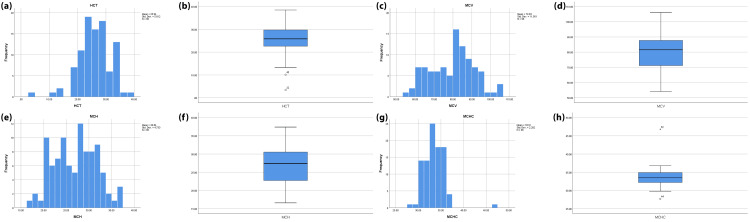
Hematological indices (HCT, MCV, MCH, and MCHC) showing no significant correlation with age or gender. (a, b) Histogram and boxplot of hematocrit (HCT) values. (c, d) Histogram and boxplot of mean corpuscular volume (MCV). (e, f) Histogram and boxplot of mean corpuscular hemoglobin (MCH). (g, h) Histogram and boxplot of the mean corpuscular hemoglobin concentration (MCHC). Histograms illustrate the frequency distribution, while boxplots show the median, interquartile range, and outliers for each parameter.

The mean red blood cell (RBC) and hemoglobin (Hb) indices in the elderly group (3.0 and 8.0, respectively) are less than the mean RBC and Hb in the young adult group (3.7 and 9.3, respectively), and a strong negative correlation was found between age and RBC and Hb (Figure 1). On the other hand, no significant differences were found between age and other hematological indices (Figure 2).

Effect of gender on the hematological picture

No major differences were noted in the RBC counts between the males and females in this study; however, the mean Hb level was lower in the female group (8.4 g/dL). The mean platelet count in the male group was 340, while the mean platelet count in the female group was slightly higher (364), as illustrated in Table 4.

**Table 4:** The mean hemtological indices of the patients included in the study in relation to their gender. HCT: Hematocrit; Hb: Hemoglobin; MCH: Mean corpuscular hemoglobin; MCHC: Mean corpuscular hemoglobin concentration; MCV: Mean corpuscular volume; RBC: Red blood cells

Female	Male	Variables
3.3	3.5	RBC
8.4	9	Hb
25.1	26.4	HCT
81.2	78.8	MCV
28.4	26.6	MCH
33.0	33.8	MCHC
364	340	Platelet

**Figure 3:**
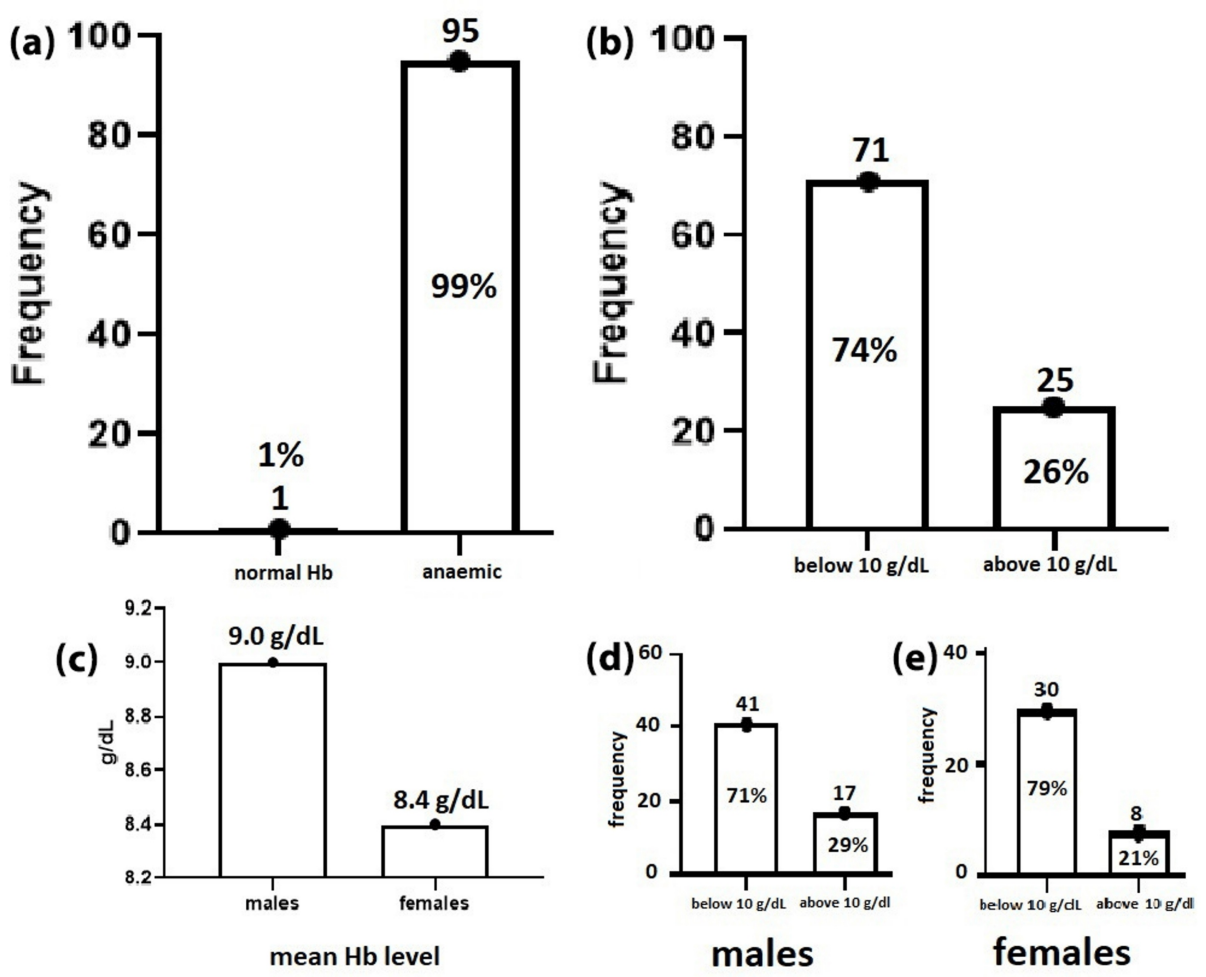
Distribution of patients with normal hemoglobin (Hb) levels and patients with anemia among study population. (a) Overall classification of participants based on their hemoglobin levels, showing 1% (n = 1) had normal Hb levels and 99% (n = 95) were anaemic. (b) Distribution of participants <10 g/dL or >10 g/dL, with 74% (n = 71) below and 26% (n = 25) above this level. (c) Comparison of mean Hb levels between males (9.0 g/dL) and females (8.4 g/dL). (d) Male participants: 71% (n = 41) had Hb <10 g/dL, while 29% (n = 17) had Hb >10 g/dL. (e) Female participants: 79% (n = 30) had Hb <10 g/dL, while 21% (n = 8) had Hb >10 g/dL.

Most of the studied patients (99%) were found to be anemic, with the majority (74%) suffering from severe anemia (Hb below 10 g/dL), while only 26% were moderately affected by the disease (Hb above 10 g/dL). SCD severity was also found to be higher (8%) among females when compared to males (79% vs. 71%), as shown in Figure 3.

**Figure 4:**
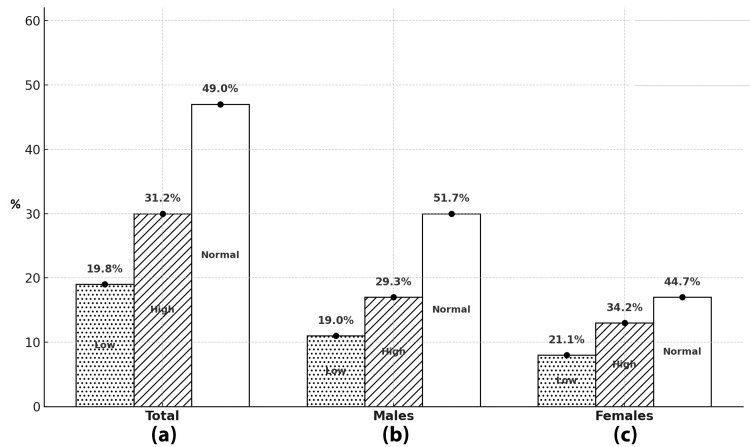
The percentage of normal and abnormal platelet counts among studied SCD patients and differences in these percentages among males and females. Distribution of platelet counts among participants categorized as low, high, and normal. (a) Overall distribution: 19.8% had low platelet, 31.2% had high platelet, and 49.0% had normal platelet levels. (b) Among males: 19.0% had low, 29.3% had high, and 51.7% had normal platelet levels. (c) Among females: 21.1% had low, 34.2% had high, and 44.7% had normal platelet levels. SCD: Sickle cell disease

In addition, we found that about half of the studied SCD patients (51%) had abnormal platelet counts, with high abnormal counts representing approximately 30% and abnormal low platelet counts of about 20%, with only a slight difference in the percentages of these abnormalities between males and females, as illustrated in Figure 4.

## Discussion

SCD is a monogenic disorder with a wide array of morbidity and mortality complications that negatively affect the quality of life of the patients affected by it [11,12]. This study illustrates the hematological characteristics of SCD patients in the Al Ahsa region in the eastern province of Saudi Arabia. The study finds several differences in the hematological picture between males and females in the study and between different age groups.

Unlike previous studies that show that the global SCD prevalence was slightly larger in females when compared with males (3.90 vs. 3.84 million, respectively) [1], most of the patients in the current study were males. However, these differences could be due to the relatively small size of the sample involved in our study. Most of the patients in this study were also young, with a mean age of 33, which is in accordance with findings from a previous study done by Bin Zuair et al. in Saudi Arabia, who performed similar retrospective studies. However, unlike the current study, most of the patients were from the southern area, which could explain the slightly younger age mean of 31 years [5]. Furthermore, it was noted in the current analysis that the increase in the age of the patients correlates negatively with the value of Hb and RBCs, which is evident by the difference in these values between younger adults and elderly individuals in this study, as evident by Pearson correlation (Tables 2-3).

Previous studies have demonstrated a difference between the clinical picture of patients with sickle disease in the eastern and western regions, with the eastern province patients having fewer complications and a milder form of the disease. Furthermore, they were shown to have a higher level of Hb and a lower level of platelet counts when compared with the patients from the western province [13]. This could be due to the high level of fetal Hb as reported in previous studies in the eastern province [7].

These findings were in accordance with the findings in our current study, as most of the patients suffer from a relatively mild form of anemia and thrombocytopenia, with female patients showing a slightly higher level of platelet counts when compared to males (Figure 4). In contrast, the patients from western regions in Saudi Arabia have a more severe form of the disease, with a unique set of complications such as dactylitis and stroke, which is more common in the patients in this region than in the patients from eastern regions, such as Al Ahsa [14].

A previous study was done by Udezue et al. in the eastern region of Saudi Arabia to analyze gender-related differences associated with the admitted SCD patients [15]. Here, it was noted that the males constituted 53% of the stability unit admissions. Moreover, the mean age was significantly lower than that of our current study (24.8 vs. 33 years). The difference in the mean age of admission could be due to the difference in the level of healthcare available across the years. No major differences were noted in the mean Hb in males and females between this study and our current study (9.5 vs. 9 g/dL for males and 9 vs. 8.4 g/dL for females). Furthermore, it was noted in the current study that most of the participants had an Hb level below 10 g/dL, with females showing a higher tendency toward anemia (Tables 2-3).

While this study provides valuable insight into the hematological characteristics of adult SCD patients in the Al Ahasa region, it is still limited by the relatively small size of the included sample and the nature of the single-center design of the study. However, as the included sample represents data from patients admitted over relatively large durations, we believe the overall results will provide significant addition to the body of knowledge related to the SCD in Saudi Arabia.

## Conclusions

Our study gives a detailed analysis of the hematological characteristics of SCD in the Al Ahsa region, Saudi Arabia, highlighting notable trends and differences across age and gender. The findings show that most patients in the region experience mild anemia and abnormal platelet counts, with females being slightly higher in prevalence of severe anemia and increased platelet counts compared to males. Age was inversely correlated with Hb and RBC levels, suggesting that older patients are more likely to experience severe disease progression.

The study also aligns with prior research indicating regional variations in SCD severity within Saudi Arabia, with patients from the eastern region, including Al Ahsa, generally experiencing milder forms of the disease compared to those in the western region. While these findings contribute to understanding the hematological and demographic aspects of SCD in this specific region, limitations such as the relatively small sample size and single-center design highlight the need for further research involving larger, multi-center cohorts to enhance generalizability and provide a comprehensive understanding of SCD in Saudi Arabia.
